# Absorption coefficients data of lead iodine perovskites using 14 different organic cations

**DOI:** 10.1016/j.dib.2019.104636

**Published:** 2019-10-07

**Authors:** César Tablero Crespo

**Affiliations:** Instituto de Energía Solar, E.T.S.I. de Telecomunicación, Universidad Politécnica de Madrid, Ciudad Universitaria s/n, 28040 Madrid, Spain

**Keywords:** Perovskites, Optical properties, Photovoltaic, Semiconductors

## Abstract

This Data article presents the absorption coefficients of Lead Iodine perovskites using 14 different organic cations. In addition, the absorption coefficients have been split into inter-atomic species components in order to quantify all of the contributions. For more details on the methodology, interpretation and discussion, refer to the full length article entitled “Effect Of the organic cation on the optical properties of lead iodine perovskites”. https://doi.org/10.1016/j.solmat.2019.110022 Data may be useful for future research, and to identify the contribution of different species to the absorption.

**Table:** Specifications Table

Subject	Energy
Specific subject area	Renewable Energy, Sustainability and the Environment
Type of data	Table and Figures
How data were acquired	They have been obtained from first principles in two steps: (i) the electronic properties are obtained from first-principles calculations based on density-functional theory, and (ii) the optical properties were obtained from the imaginary part of the dielectric function.
Data format	Raw and analysed
Experimental factors	The imaginary part of the dielectric function was calculated within the random phase approximation. The absorption coefficients and other optical properties were obtained from the imaginary part of the dielectric function using the Kramers-Kronig relationships. Later, they were split into inter-species, intra-species, 3-species and 4-species contributions
Experimental features	The absorption coefficients of Lead Iodine perovskites using 14 different organic cations were obtained using first principles calculations. Later, they were split as a many-specie expansion.
Data source location	Instituto de Energía Solar, E.T.S.I. de Telecomunicación, Universidad Politécnica de Madrid, Ciudad Universitaria s/n, 28040 Madrid, SPAIN.
Data accessibility	Repository name: zenodo Data identification number: 10.5281/zenodo.3335833 Direct URL to data: https://doi.org/10.5281/zenodo.3335833
Related research article	César Tablero Crespo, Effect Of the organic cation on the optical properties of lead iodine perovskites, Solar Energy Materials and Solar Cells, https://doi.org/10.1016/j.solmat.2019.110022.

**Table:** 

**Value of the data** • The total absorption coefficients can serve as reference for experimental results in perovskites with different organic cations. • The inter-atomic species components of the absorption coefficients allows to analyze the optical characteristics that the substitution of Pb by another element should satisfy to maintain a high absorption capacity. • The total absorption coefficients can be used to obtain efficiencies of solar cells.

## 1 Data

**Fig. 1:**
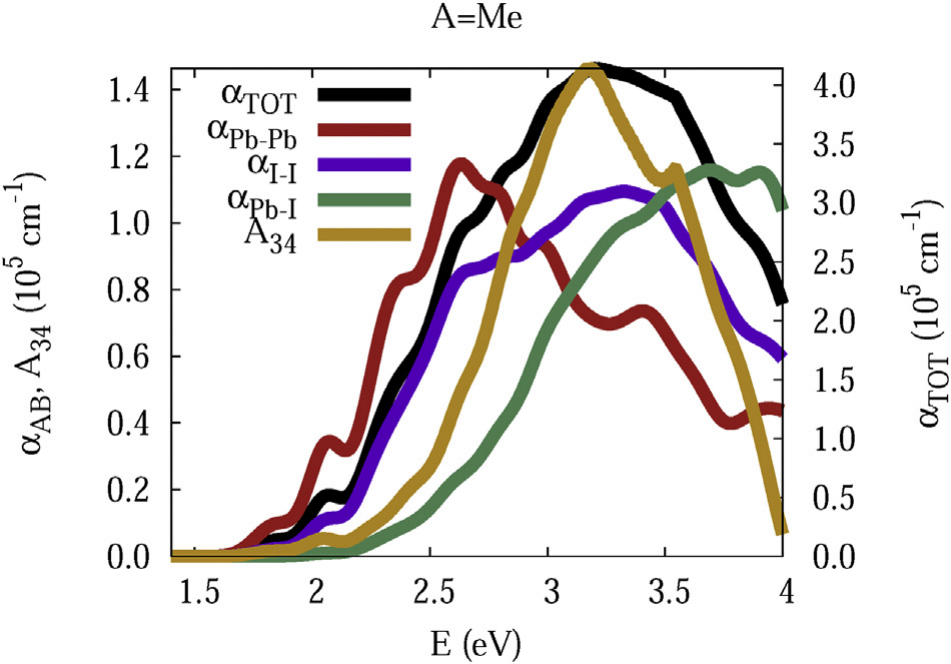
${\text{APbI}}_{3}$total absorption coefficient$\alpha_{\text{TOT}}$ (right *y* axis) for A = Me. The most important intra- and inter-specie $\alpha_{\text{Pb}-\text{Pb}}$, $\alpha_{\text{I}-\text{I}}$, $\alpha_{\text{Pb}-\text{I}}$, and the 3 and 4-species contributions ($A_{34}$) are represented in the left *y* axis.

**Fig. 2:**
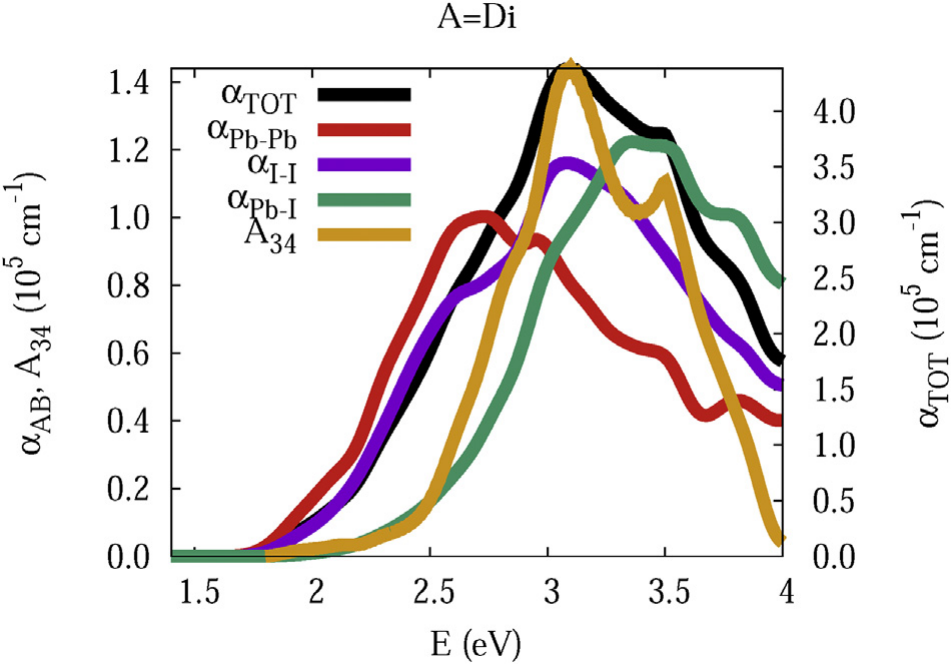
Same legend as that in Fig. 1, but for A = Di.

**Fig. 3:**
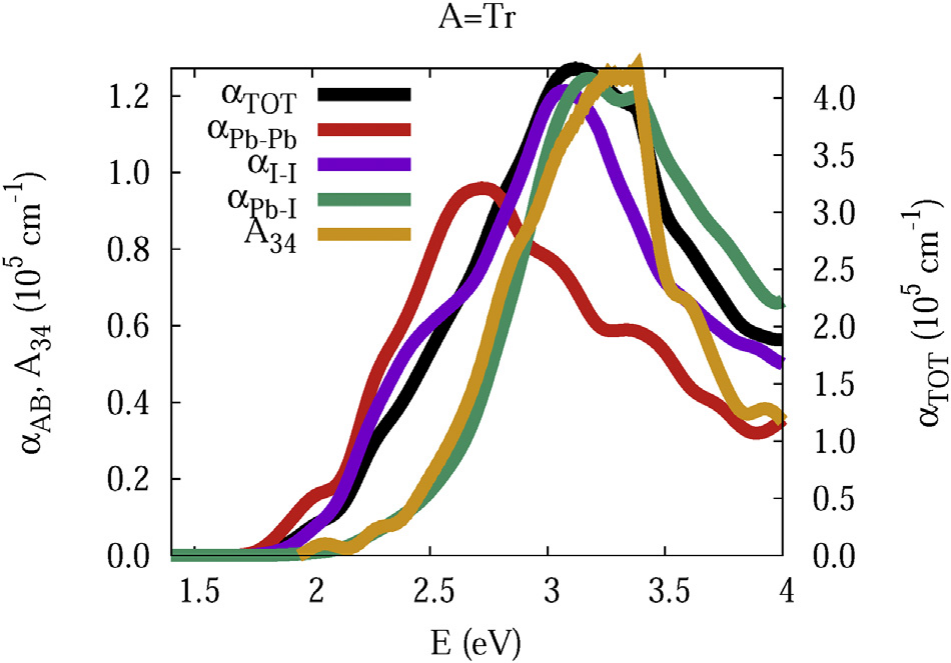
Same legend as that in Fig. 1, but for A = Tr.

**Fig. 4:**
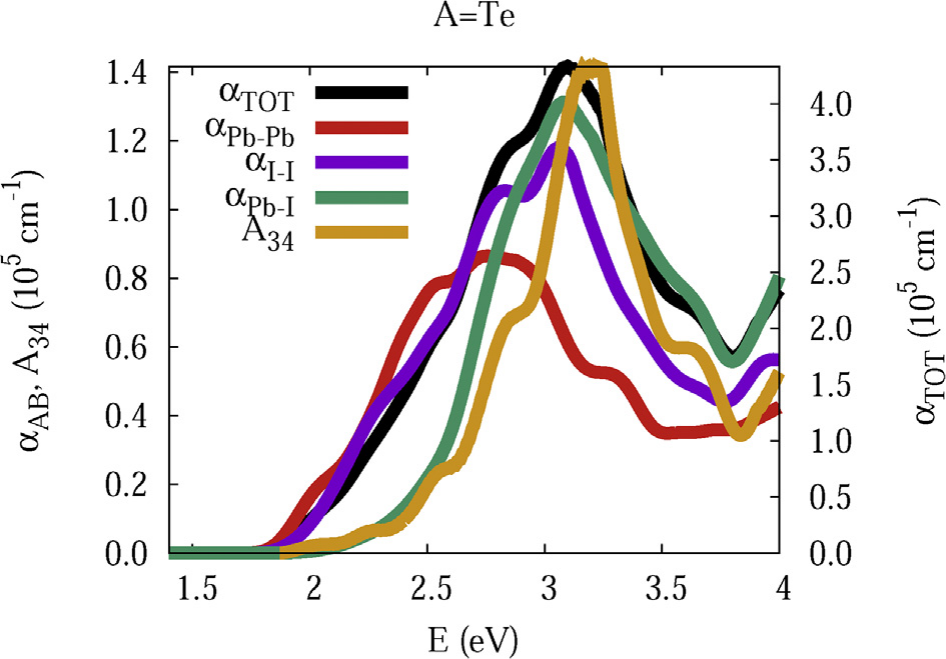
Same legend as that in Fig. 1, but for A = Te.

**Fig. 5:**
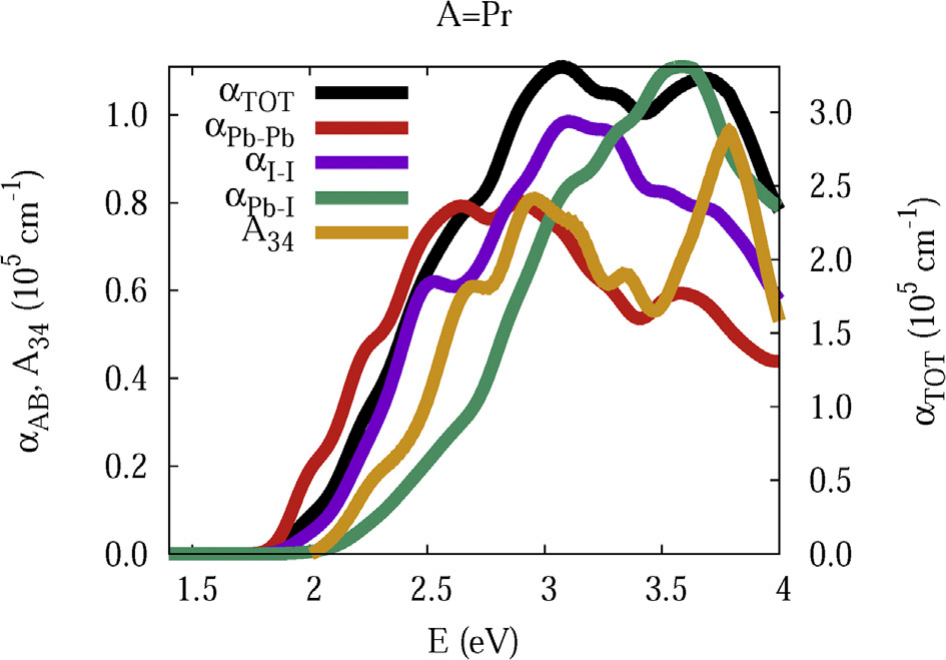
Same legend as that in Fig. 1, but for A = Pr.

**Fig. 6:**
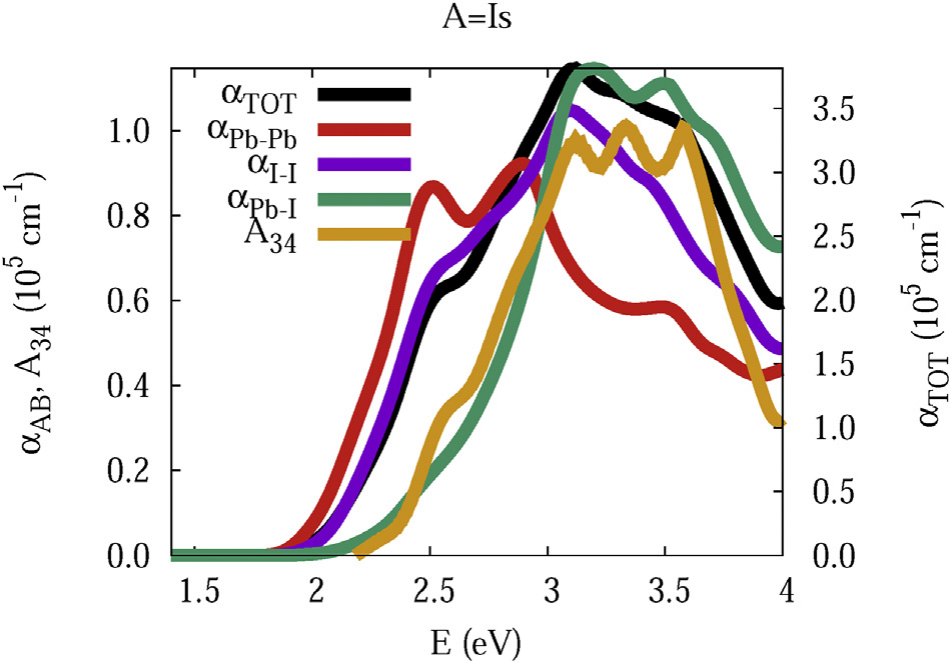
Same legend as that in Fig. 1, but for A = Is.

**Fig. 7:**
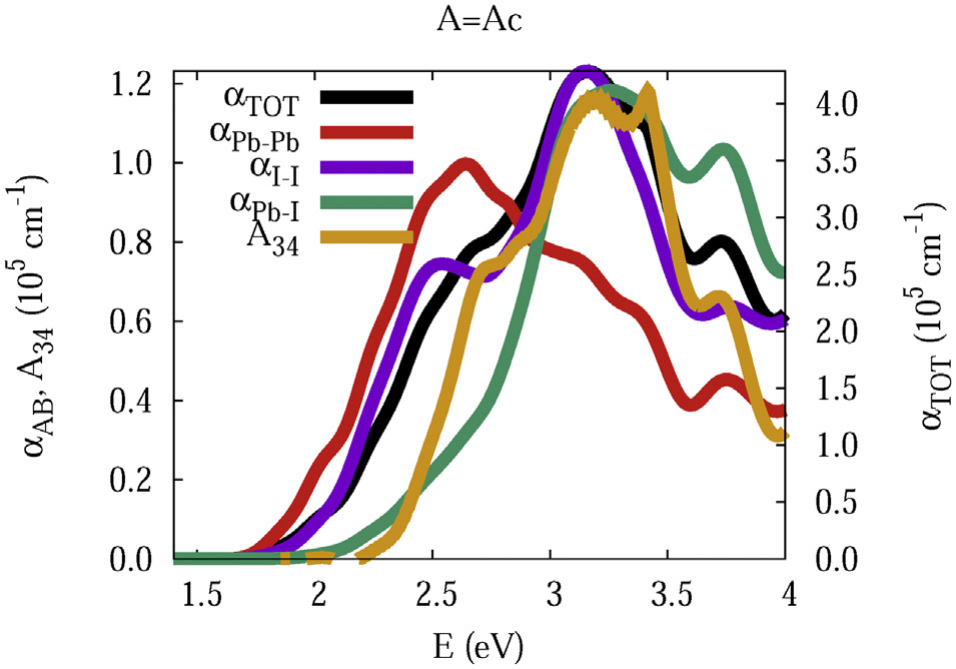
Same legend as that in Fig. 1, but for A = Ac.

**Fig. 8:**
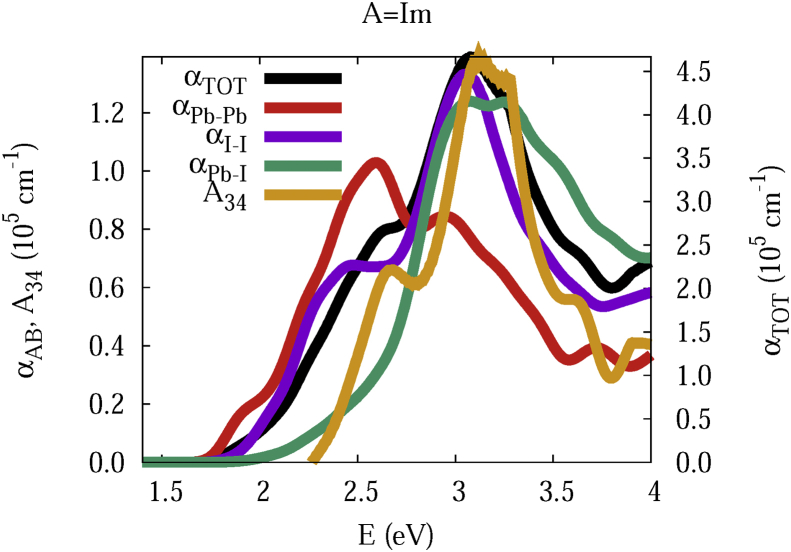
Same legend as that in Fig. 1, but for A = Im.

**Fig. 9:**
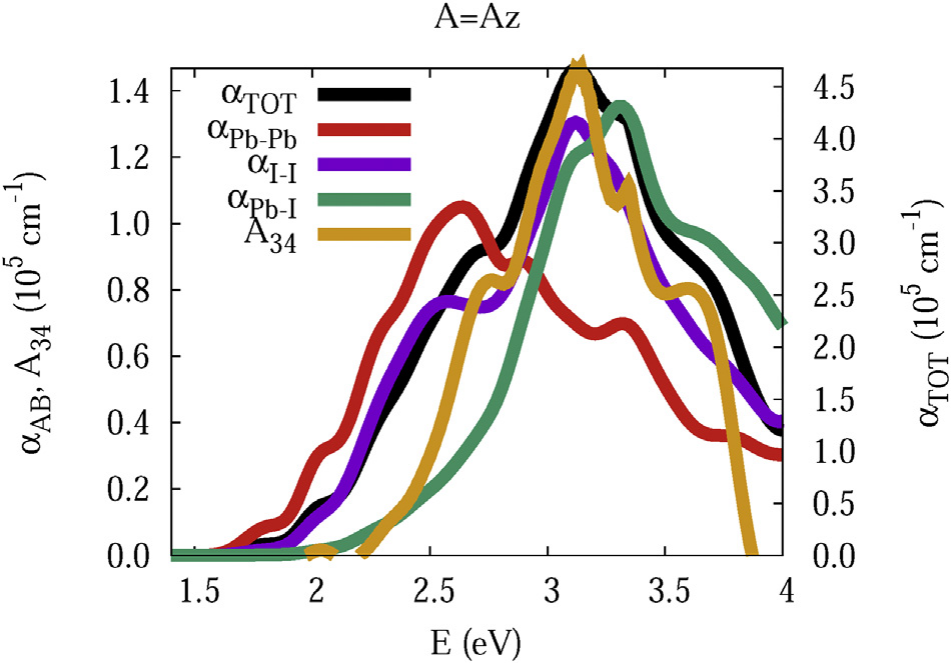
Same legend as that in Fig. 1, but for A = Az.

**Fig. 10:**
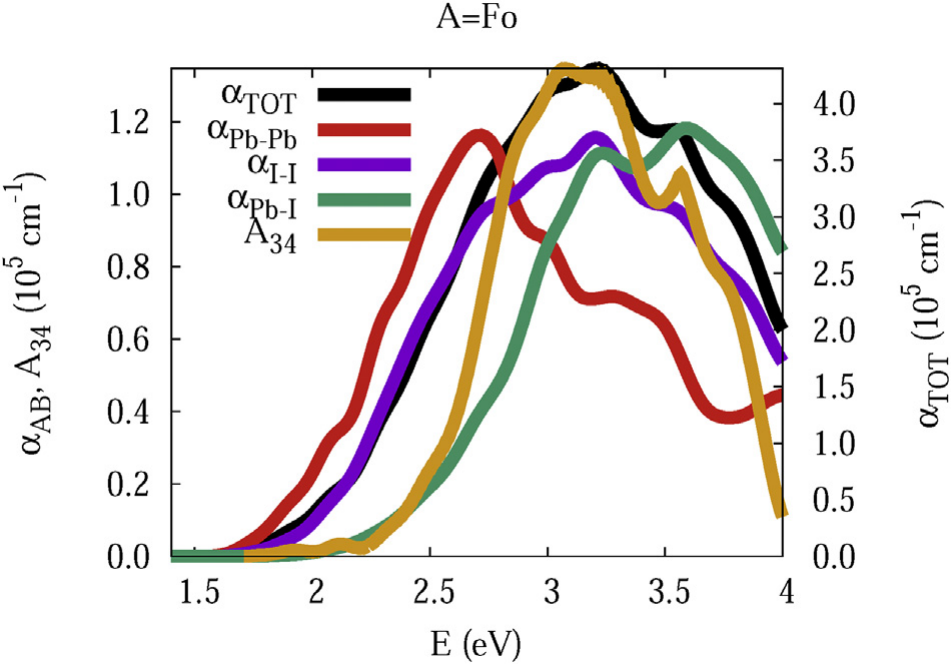
Same legend as that in Fig. 1, but for A = Fo.

**Fig. 11:**
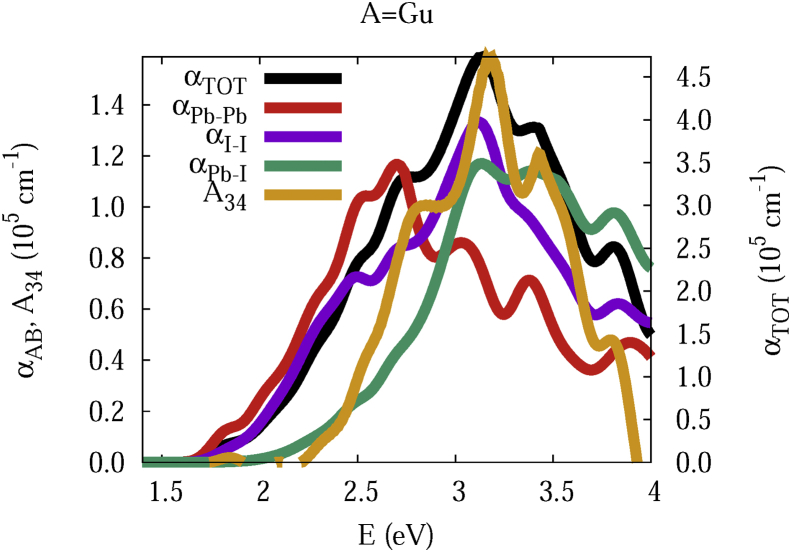
Same legend as that in Fig. 1, but for A = Gu.

**Fig. 12:**
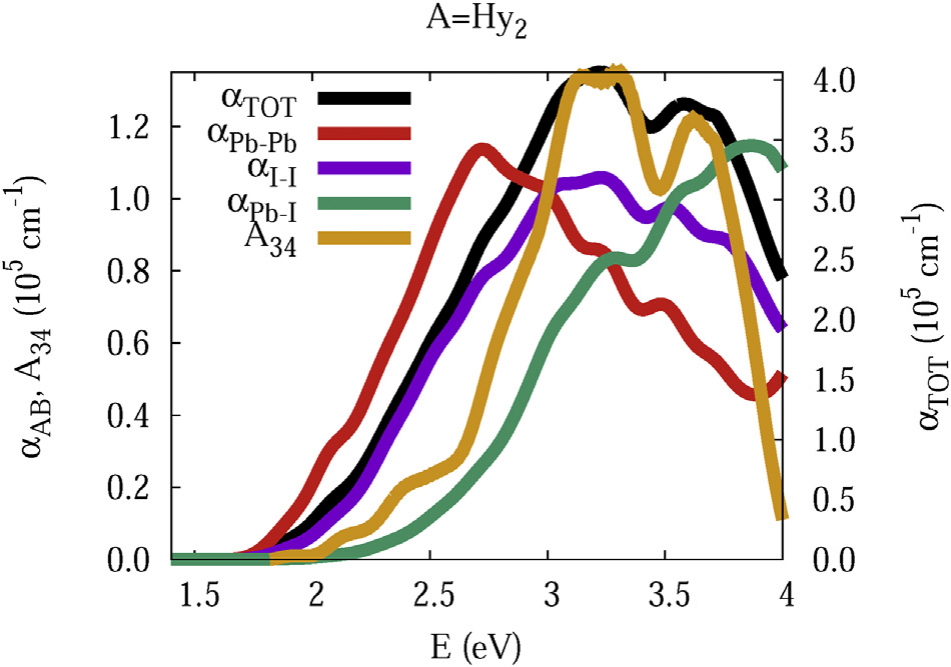
Same legend as that in Fig. 1, but for A = ${\text{Hy}}_{2}$.

**Fig. 13:**
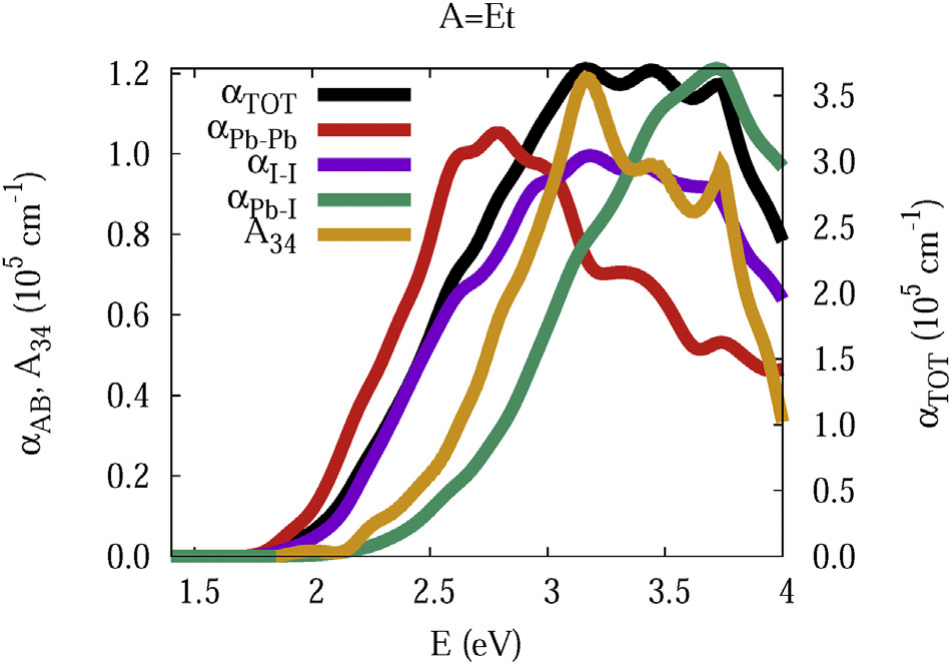
Same legend as that in Fig. 1, but for A = Et.

**Fig. 14:**
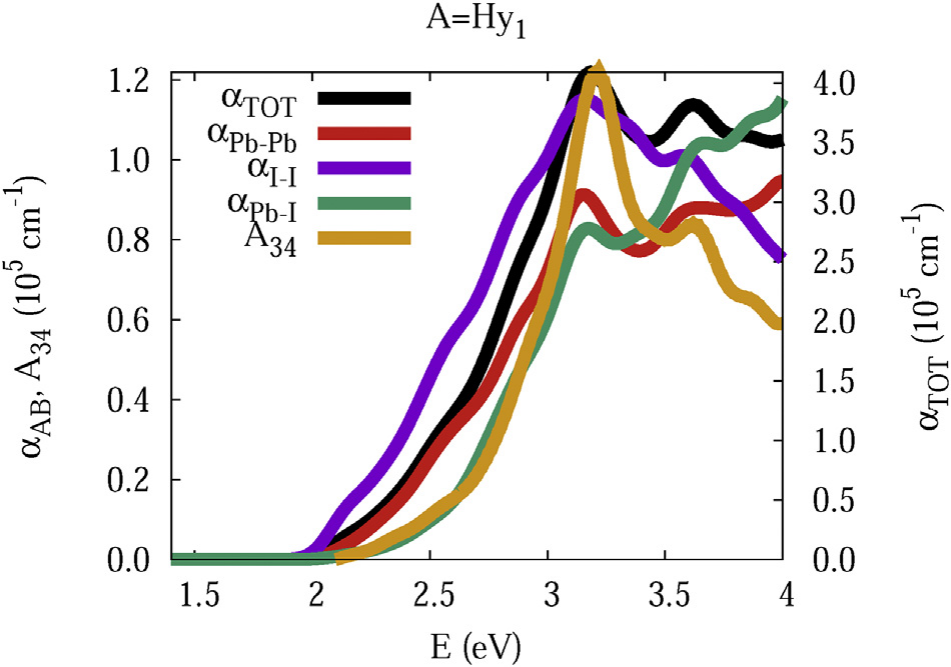
Same legend as that in Fig. 1, but for A = ${\text{Hy}}_{1}$.

The data and figures shown below represent the total and split absorption coefficients of 14 organic cations of lead iodine perovskites. The total absorption coefficients $\alpha_{\text{TOT}}$have been split into inter-atomic species components $\alpha_{\text{TOT}}=A_{12}+A_{34}$. The first term$A_{12}=\sum_{A}\sum_{B}\alpha_{AB}$ involves intra-species (A = B) and inter-species (A≠B) contributions, and the $A_{34}$ term involves 3-species (A≠B≠C) and 4-species (A≠B≠C≠D) contributions. It allows to quantify and split into contributions other properties related to the absorption of radiation. In Fig. 1, Fig. 2, Fig. 3, Fig. 4, Fig. 5, Fig. 6, Fig. 7, Fig. 8, Fig. 9, Fig. 10, Fig. 11, Fig. 12, Fig. 13, Fig. 14 $\alpha_{\text{Pb}-\text{Pb}}$ and$\alpha_{\text{I}-\text{I}}$are intra-species, $\alpha_{\text{Pb}-\text{I}}$is a inter-species, and $A_{34}$the 3- and 4-species contributions to the total absorption coefficient $\alpha_{\text{TOT}}$. The datafiles were deposited at *zenodo* repository (https://doi.org/10.5281/zenodo.3335833), and they contains the raw date corresponding to the total absorption coefficients for the 14 organic cations described in Table 1.

**Table 1:** Abbreviations used for organic cations.

Cation	abbreviation
Methylammonium	Me
Dimethylammonium	Di
Trimethylammonium	Tr
Tetramethylammonium	Te
Ethylammonium	Et
Propylammonium	Pr
Isopropylammonium	Is
Acetamidinium	Ac
Imidazolium	Im
Azetidinium	Az
Formamidinium	Fo
Guanidinium	Gu
Hydroxylammonium	${\text{Hy}}_{1}$
Hydrazinium	${\text{Hy}}_{2}$

## 2 Experimental design, materials, and methods

The methodology, interpretation and discussion are described in reference [1]. In Table 1 are shown the 14 organic cations (A) for the lead iodine perovskites APbI_3_, and in Fig. 1, Fig. 2, Fig. 3, Fig. 4, Fig. 5, Fig. 6, Fig. 7, Fig. 8, Fig. 9, Fig. 10, Fig. 11, Fig. 12, Fig. 13, Fig. 14 the total and split absorption coefficients.

The data has been obtained according to the following process:

(i) The electronic properties were obtained from first-principles calculations based on density-functional theory.
(ii) Using the energies and the occupation of the states obtained previously, and additionally calculating the transition probabilities, the optical properties were obtained from the imaginary part of the dielectric function within the random phase approximation.
(iii) From the dielectric function, and using the Kramers-Kronig relationships, the total absorption coefficients were obtained.
(iv) The total absorption coefficients were split into inter-species, intra-species, 3-species and 4-species contributions according to the process described in the previous section.

A remarkable feature of the previous figures is that almost all organic lead iodine perovskites absorption coefficients are qualitatively similar. For energies close to the bandgaps, the main contribution to the absorption coefficient is from the Pb–Pb intra-species transitions. With the increase in the photon energy above the energy bandgaps, the I–I and Pb–I contributions, and the contributions of 3 and 4 species begin to be as important as the Pb–Pb contribution. The exception is for the ${\text{Hy}}_{1}$cation with a larger energy bandgap than the rest of the perovskites. Note that the organic cation atoms almost do not contribute to the intra- and inter-species terms. In the 3 and 4 species terms are included the organic cation contributions. Therefore, summarizing the data: (i) the absorption properties do not vary considerably, except for the ${\text{Hy}}_{1}$cation; (ii) the organic cations do not directly contribute to the optical properties via intra- and inter-species terms, but indirectly through of the 3 and 4 species terms for energies above the energy bandgap.
